# Exploring negative experiences in psychotherapy using an NLP approach on online forum data

**DOI:** 10.1038/s44184-025-00172-4

**Published:** 2025-11-07

**Authors:** Tobias Steinbrenner, Christopher Lalk, Alin Kabjesz, Drin Ferizaj, Juan Segundo Pena Loray, Flavio Iovoli, Julian Rubel

**Affiliations:** 1 Department of Psychotherapy Research and Clinical Psychology, Osnabrueck University, Osnabrück, Germany; 2 Department of Geriatrics and Medical Gerontology, Charité - Universitätsmedizin, Berlin, Germany

**Keywords:** Adverse effects, Human behaviour, Health services

## Abstract

Negative experiences with psychotherapy are common, affecting 3–25% of patients. However, their causes remain underexplored despite their substantial impact on therapy outcomes. Online forums provide unique insights into patients’ concerns due to their anonymity. We collected and anonymized forum posts and used a large language model to identify psychotherapy dissatisfaction. Human raters validated the outputs. To identify and analyze themes, we applied clustering, topic modeling, sentiment analysis, and classification based on an existing meta-analytic framework. In total, we extracted 28,079 text passages reflecting dissatisfaction. Clustering yielded 55 subthemes, covering therapist misbehavior, negative treatment effects, poor alliance, treatment mismatch, and healthcare-related frustrations, extending existing taxonomies. Our NLP-based, mixed-methods approach highlights dissatisfaction as both frequent and multifaceted, surfacing themes often overlooked in traditional research, such as structural barriers and lasting psychological consequences. These findings expand previous frameworks and underscore the need for better recognition of negative therapy experiences.

## Introduction

Despite the broad evidence suggesting that psychotherapy is effective on average, still 31–40% do not benefit significantly from treatment^1^. In addition, between 3% and 25% report negative experiences or dissatisfaction with their psychotherapy^2–5^. Some negative experiences in psychotherapy can be worked through together by therapists and patients, resulting in positive effects. A therapist can react to unforeseen changes, requirements, and behaviors of patients and adapt their therapeutic interventions individually to the patient^6^. However, what happens when these negative experiences remain unrecognized?

Patient dissatisfaction is associated with a number of adverse consequences, including premature termination of treatment^7,8^, treatment non-response, and symptom deterioration^9^. However, it should be noted that not all non-improved patients report dissatisfaction with their psychotherapy^10,11^ and that subjective evaluations of therapy may not always align with objective indicators of clinical improvement^12^. Dissatisfaction with psychotherapy can lead to ineffective interventions by therapists and a deterioration of the therapeutic alliance^11^, as dissatisfaction can hinder open communication, leading therapists to apply interventions that are not appropriate for the respective patient’s evolving needs^13,14^. Patients may develop a tendency to overly rely on themselves in managing distress, potentially avoiding professional help even when needed^14^. Previous research has found a negative correlation between satisfaction with prior therapy and doubts about its effectiveness^15^. This dissatisfaction can decrease the likelihood that patients will seek help in the future.

Despite the importance of negative experiences in therapy, research suggests that they are less frequently reported than positive ones^16–19^. This pattern may indicate that positive experiences generally prevail in therapy, although it has been suggested that clients hesitate to disclose negative experiences^20^. One key issue is the discrepancy between therapists’ perceptions and patients’ actual experiences^13,21^. Therapists often misjudge their patients’ reactions to interventions^22^, underestimate negative outcomes^11^, or underestimate the impact of patient dissatisfaction^7^. At the same time, patients frequently refrain from expressing dissatisfaction, either because they struggle to articulate subtle feelings of discontent^3,14^ or because they fear upsetting their therapist. Even when explicitly invited to share negative feedback, clients often remain reluctant to do so^23,24^.

Despite the difficulties in studying psychotherapy dissatisfaction, research into negative experiences in psychotherapy has highlighted multiple factors contributing to patients’ dissatisfaction and poor outcomes. A qualitative meta-analysis categorized these experiences into four overarching clusters: (1) Therapists’ misbehavior, (2) Hindering aspects of the relationship, (3) Poor treatment fit, and (4) Negative impacts of treatment^20^. Other work proposed a distinction between unwanted events caused by psychotherapy (e.g., side effects, malpractice) and those unrelated to therapy^25^. A synthesis of qualitative studies and client testimonies suggested eight overarching domains of adverse process factors, including contextual, relational, client, and therapist-level contributors^26^. Client-identified hindering experiences include emotional disconnection, lack of guidance, and feeling overwhelmed^27^. In addition to these meta-analyses, individual studies have explored patients’ dissatisfaction stemming from therapy-related issues, poor therapeutic relationships, and unmet expectations^5^. Dissatisfaction has also been linked to unaddressed patient needs and perceived therapist incompetence, with alliance ruptures contributing to poor outcomes^28^. One study highlighted that although malpractice indicators were rare, 16.8% of participants felt violated by their therapist’s statements^29^. This body of literature underscores the complex nature of psychotherapy dissatisfaction, which arises from a combination of interpersonal, contextual, and treatment-related factors.

Despite the valuable insights from previous studies, important gaps in understanding psychotherapy dissatisfaction persist. Many studies focus on small, specific samples, such as certain age groups or clinical conditions. While this approach is useful for examining specific reasons for dissatisfaction, it limits the generalizability of findings. One meta-analysis found highly heterogeneous reasons for dissatisfaction, with no single meta-category occurring in >65% of included studies^20^. Furthermore, research on negative effects under naturalistic conditions remains limited, and more detailed data are needed to understand their prevalence and persistence across different treatments and settings^20,29^. While qualitative research has provided valuable insights into these dynamics, quantitative research on negative experiences is scarce.

Since internal states cannot be observed directly, researchers must rely on patients’ willingness to report them; external circumstances can influence this willingness, leading to socially desirable responses. Consequently, negative experiences are disclosed far less often than positive ones, indicating a reporting bias^11,24,27,30^. When disclosure barriers are removed, however, patients describe a broad spectrum of adverse effects^20^, highlighting the need for large-scale methods that can capture these accounts more accurately.

Anonymous online forums mitigate much of this bias: the anonymity and accessibility of online forums facilitate self-disclosure, where individuals may express themselves more openly than in face-to-face settings^31,32^. Beyond anonymity, recent research highlights additional factors that promote disclosure, including asynchronicity, shared social identity, and reciprocity^33,34^. Studies on suicidality and substance-use recovery have shown that users disclose sensitive experiences more freely in online forums than in conventional surveys^35,36^. Thus, online forums might reveal issues that would otherwise remain unspoken in therapy or traditional research contexts.

Posts can be analyzed using natural language processing (NLP), the computational modelling and analysis of human language. NLP has already been used in mental-health research to detect suicidality, depressive language, or treatment-seeking intent in social-media text^35,37^ and, on a smaller scale, to analyze psychotherapy session transcripts^38,39^. These studies typically rely on keyword lexica, classical classifiers, fine-tuned BERT models, and general-purpose large language models (LLM)^40^. Recently, an LLM-based pipeline was applied to examine therapeutic group factors in Reddit communities, employing iterative coding, code-based clustering, and human thematic analysis to detect key processes within online interactions. This study provided a proof-of-concept for the integration of data-driven analyses of a large text corpus within a theoretical framework under human supervision^41^. To our knowledge, no work has yet combined a large-language-model extractor with unsupervised density-based clustering and human validation to map patient-reported processes, such as psychotherapy dissatisfaction, at scale.

Leveraging this insight, we introduce a mixed-methods NLP pipeline that uses LLMs to extract over 28,000 text passages of psychotherapy dissatisfaction from >500,000 forum posts. We uncover their thematic structure using unsupervised clustering and topic modelling, and validate every step with trained human raters. This study aims to identify common reasons for dissatisfaction from the patient’s perspective and offers a novel approach to uncovering overlooked patterns.

## Methods

The following section describes the procedures used to collect, process, and analyze Reddit posts about negative psychotherapy experiences. We outline the steps from data collection through text classification and extraction, followed by NLP-based analyses and integration with qualitative frameworks. Our approach combines automated LLM techniques with manual validation and interpretation to ensure methodological rigor and contextual relevance.

### Data collection

We collected publicly accessible Reddit posts and comments from 100 mental health-related subreddits between 2022 and 2024, as this period reflects recent and thematically relevant user experiences. Data were extracted using the Python Reddit API Wrapper (PRAW)^42^. Posts and their associated comments were only included if the post contained at least one of the following keywords: therapist, psychotherapist, psychologist, treatment, therapeutic professional, therapy, psychotherapy, dissatisfied, negative experience. The selection of keywords was kept broad to minimize search term bias for subsequent clustering. Subreddits were chosen to span a wide range of mental-health topics and therapeutic approaches, thereby reducing subreddit-specific linguistic or thematic bias. The selection process primarily targeted communities centered on mental health conditions (e.g., r/Depression, r/CPTSD, r/BingeEatingDisorder), ensuring comprehensive coverage of all ICD-10 disorder categories, as well as subreddits focusing on therapy and therapeutic approaches (e.g., r/TalkTherapy, r/CBT, and r/Psychoanalysis) to capture discussions about treatment experiences and methodologies. In total, 54,056 posts and 467,163 comments (521,219 in combination) met the inclusion criteria. Ethical considerations, de-identification, pseudonymization, and other privacy safeguards are provided in the ethical considerations section.

### Sample post information

Across the 100 subreddits, the median number of posts per subreddit was *Mdn* = 525 (*Q1* = 215; *Q3* = 848), while the median number of comments per subreddit was *Mdn* = 3489 (*Q1* = 1443; *Q3* = 6448). The median word count of a post was *Mdn* = 243 (*Q1* = 138; *Q3* = 418), whereas comments had a median word count of *Mdn* = 47 (*Q1* = 21; *Q3* = 99).

### User information

From 5362 users who reported dissatisfaction, we extracted demographic details they had disclosed in their posts and comments (see Ethics Considerations section for rationale and safeguards). The number of users was determined via unique usernames, which were pseudonymized during processing. However, multiple accounts by the same individual cannot be fully excluded. After de-identification, age information for still *n* = 1437 users was maintained. Ages were grouped into categories to prevent individual identification through exact age details. The median age category was *Mdn* = mid-twenties (*Q1* = early twenties; *Q3* = early thirties), ranging from young adult to end seventies. The most frequent categories were early twenties (*n* = 456; 31.7%), and early thirties (*n* = 265; 18.4%). Sample information on gender, education, residence, disorder categories, treatment approaches, and therapists’ gender is shown in Table 1.

**Table 1:** Sample user information

Category	*n*	%
Gender	566	100
Female	309	54.6
Male	192	33.9
Non-binary	65	11.5
Education	1178	100
Employed	408	34.6
University	393	33.4
Unemployed	276	23.4
School	87	7.4
Retired	14	1.2
Residence: continents	358	100
Europe	156	43.6
North America	146	40.8
Australia	31	8.7
Asia	15	4.2
South America	8	2.2
Africa	2	0.6
Residence: countries	358	100
United States of America	102	28.5
United Kingdom	60	16.8
Canada	39	10.9
Australia	30	8.4
Germany	24	6.7
Netherlands	14	3.9
Norway	11	3.1
Other	78	21.8
Disorder category	3415	100
Mood disorders	1349	39.5
Anxiety disorders	1191	34.9
Neurodevelopmental disorders	940	27.5
Personality disorders	655	19.2
Trauma disorders	626	18.3
Eating disorders	373	10.9
Obsessive-compulsive and related disorders	367	10.7
Treatment approach	1777	100
CBT	831	46.8
Psychodynamic therapy	196	28.7
Psychoanalysis	103	19.0
Systemic therapy	9	0.5
EMDR	510	28.7
DBT	338	19.0
Internal family systems	228	12.8
Therapists’ gender	2782	100
Female	2137	76.8
Male	579	20.8
Non-binary	67	2.4

Text passages from a total of 5362 users were analyzed. Since data for each category were available only from subsamples (based on self-reports), the sample sizes for these subsamples are provided. For residence, disorder category, and treatment approach, only the most frequently reported categories are presented. Specifically, 3415 individuals self-reported mental illnesses. For each participant, up to three diagnoses were recorded, one primary diagnosis and up to two comorbidities, resulting in a total of 5791 reported mental illness categories. The numbers in the table indicate the count of users reporting each respective disorder category. 1777 users self-reported their treatment approach or approaches. The numbers in the table reflect the number of users for each approach. Treatment methods such as DBT and EMDR were counted individually and not grouped under broader categories. The users represented 36 different countries. Educational status was determined based on the users’ posts regarding their current educational level.

### Data preprocessing and building chunks per user

We first sorted and aggregated all posts and comments chronologically for each user to provide the LLM with contextual information for classification. This approach ensured that references to earlier posts and comments by the same user (e.g., when a comment refers to a therapist as “she” without explicitly mentioning the profession) remained interpretable. Without this aggregation, relevant contextual information would not be considered. For the purpose of this study, we define a chunk as a contiguous sequence of a user’s posts and comments that is processed together as a single unit by the model. Given the limited context length of the LLM, we set a maximum of 2000 tokens (basic text units processed by the model) per chunk. If this limit was exceeded, a new chunk was created. We limited the number of chunks per user to a maximum of ten to prevent the results from being overly influenced by a few individual users.

### Defining psychotherapy dissatisfaction

We defined personal psychotherapy dissatisfaction as a feeling of being dissatisfied or discontent with one’s own psychotherapy experience. Negative experiences may be related to the therapy setting, the therapist (e.g., their characteristics or behaviors), the therapeutic process, the therapeutic approach, the patients’ behavior in therapy, stagnation or deterioration in progress, costs, or access to psychotherapy. Psychotherapy dissatisfaction is specifically related to one’s own experience in therapy. This definition guided the subsequent classification process and the extraction of relevant text passages.

### Classification

We classified whether the chunks were related to psychotherapy dissatisfaction using the gpt-4o-mini-2024-07-18, at the time of the analysis, a cutting-edge LLM offering relatively low inference duration and cost, accessed via the OpenAI API. The model was provided with specific instructions, including our definition of psychotherapy dissatisfaction (see Supplementary Note 1 for the full prompt). We set the temperature parameter to 0.0 to achieve a high level of reproducibility of the classification.

Then, we selected a stratified random sample of 1000 chunks, ensuring that all sub-forums were proportionally represented to reduce biases across different sub-forums. Of these, 50% had been pre-rated as “Yes” and 50% as “No” by the LLM. We increased the proportion of “Yes”-rated chunks within this sample, as these posts were included in the subsequent analysis, whereas “No”-rated chunks were not. A trained human rater, an undergraduate psychology student specializing in clinical psychology and psychotherapy, independently rated the sampled posts. The rater was blinded to the model’s classification and received specific instructions and training before annotation. We then calculated Cohen’s kappa to assess the agreement between human and model classifications.

### Extraction of text passages

Since the chunks did not exclusively focus on psychotherapy dissatisfaction but also covered other topics, the next step was to extract text passages, defined as coherent segments of a chunk, specifically related to psychotherapy dissatisfaction. The gpt-4o-2024-11-20 model was used to filter all text passages based on the previously defined criteria for psychotherapy dissatisfaction (see Supplementary Note 2 for the full prompt). This model offers higher accuracy, improved contextual understanding, and enhanced reasoning capabilities, but at the expense of increased computational cost and longer inference times compared to the gpt-4o-mini-2024-07-18. The same trained human rater as in the previous step independently extracted text passages from a stratified random sample of chunks, ensuring that all sub-forums were proportionally represented. The rater did not know which and how many passages had been extracted by the GPT model. We evaluated the overlap between LLM- and human-extracted passages using ROUGE (Recall-Oriented Understudy for Gisting Evaluation). ROUGE-1, ROUGE-2, and ROUGE-L assess unigram, bigram, and longest common subsequence overlap, respectively, and are widely used in summarization and information extraction tasks^43^.

### Clustering

The text passages extracted by the LLM were further processed for clustering. We applied clustering, an unsupervised learning technique, to group the text passages based on their content. First, contextual embeddings were generated using the SentenceTransformer model all-mpnet-base-v2^44^. This model is well-suited for short texts, such as our text passages, and for computing text similarities, which is essential for clustering.

Distance measures in high-dimensional spaces become less precise, which is why dimensionality reduction should be applied first to improve cluster quality. Therefore, as the second step, we reduced the dimensionality of the input embeddings using UMAP, a non-linear dimensionality reduction algorithm^45^. Third, we applied HDBSCAN^46^, a hierarchical density-based clustering algorithm designed to identify dense regions (clusters) within data. Outliers are defined as text passages that HDBSCAN did not assign to any cluster due to insufficient local density.

Fourth, we conducted an internal cluster validation using the silhouette score^47^ and the Calinski-Harabasz index^48^, complemented by a brief manual review, to assess the quality of the identified clusters and compare different clustering solutions (see Supplementary Table 1 for parameter settings). Since no ground truth labels exist for the construct of psychotherapy dissatisfaction, an external validation was not possible. Additionally, we briefly manually reviewed the identified clusters. After considering the two indices and the brief manual review, we decided on the best-fitting solution.

Fifth, two raters (a trained psychology student who had also participated in earlier rating tasks, and a PhD candidate in psychotherapy research) labeled the identified clusters by manually reading the associated text passages of each cluster through inductive thematic analysis^49^. In cases of uncertainty, the raters referred to the topics generated through BERTopic^50^, discussed their labels, re-examined the associated text passages, and reached a consensus on the final cluster names.

### Topic modeling

Topic modeling aims to identify latent themes in text data and organize them into interpretable topics. BERTopic generates coherent topic representations by assigning one topic to each cluster using a class-based TF-IDF procedure^50^. TF-IDF, the product of term frequency and inverse document frequency, highlights important n-grams within a topic. In BERTopic, embeddings are first created, then reduced in dimensionality, and subsequently clustered. Since we had already completed these steps with optimized parameters in clustering, we directly applied the newly generated and extracted topics, with each topic consisting of n-grams ranging from unigrams to trigrams. Topic modeling was performed to gain a deeper understanding of the respective clusters and has already been successfully employed for psychotherapy research^39^.

### Pre-determined clusters and meta-categories

We aimed to assess the extent to which the newly generated clusters align with the categories identified in previous studies. To achieve this, we used the clusters and meta-categories from a qualitative meta-analysis^20^, which consists of 21 meta-categories grouped into four clusters. In the following, we will call these pre-determined clusters and pre-determined meta-categories. The four pre-determined clusters were named Therapists’ Misbehavior, Hindering Aspects of the Relationship, Poor Treatment Fit, and Negative Impacts of Treatment.

Two authors independently categorized the newly generated clusters into the pre-determined meta-categories. Then, they compared their assignments, and in cases where they disagreed, they re-evaluated the assignments through discussion to reach a final decision. If no consensus was reached, the cluster was assigned to a separate “no fit” category. After all clusters were categorized, the clusters in the “no fit” category were re-examined to search for potential overlaps that could lead to the formation of a new category.

### User-level analysis

We analyzed the presence and variety of patient contributions across clusters, focusing on the number and variety of dissatisfaction reasons reported by individual users.

To achieve this, we calculated the average number of text passages per individual and the average number of clusters each user contributed to. Additionally, we examined the distribution of the pre-determined clusters across individual users and assessed the frequency of cluster co-occurrences within users.

### Sentiment analysis

**Fig. 1:**
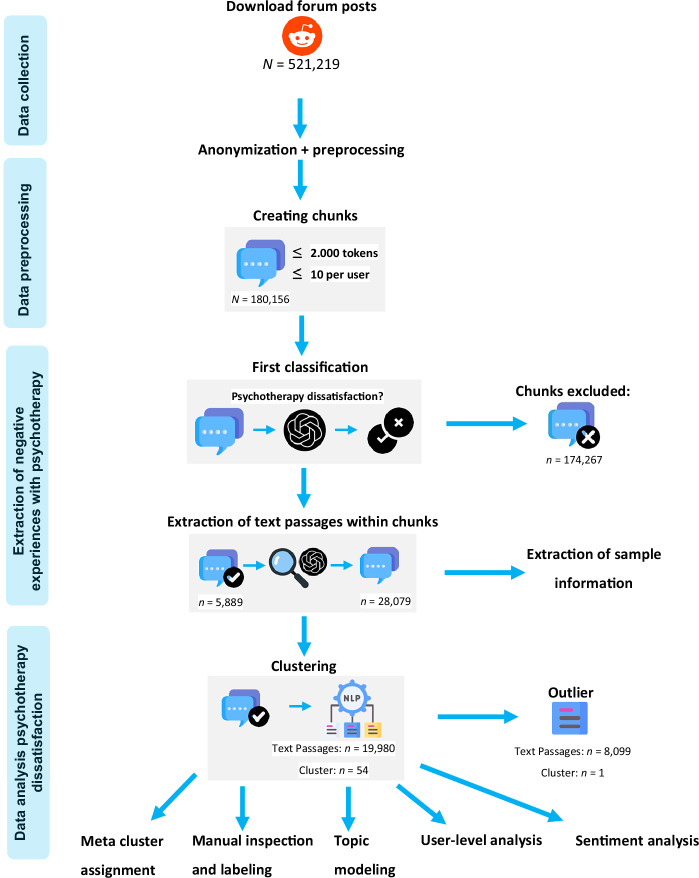
NLP-mixed-methods pipeline for extracting and analyzing psychotherapy dissatisfaction. Blue rounded boxes represent preprocessing steps, while icons indicate the respective task. NLP refers to natural language processing. Forum posts were anonymized and aggregated into chunks, defined as consecutive aggregated posts and comments from the same Reddit user. Tokens are the basic text units processed by the model, typically corresponding to short words or word fragments. Chunks classified as relevant were further divided into text passages. A text passage is a segment within a chunk that specifically relates to dissatisfaction with psychotherapy. Subsequent steps included meta cluster assignment, manual inspection and labeling of clusters, topic modeling, user-level analysis, and sentiment analysis. Icons sourced from Flaticon.com.

We aimed to identify which of the newly generated clusters and the pre-determined clusters were associated with the strongest negative affect. To measure negative, neutral, and positive affect, we used the roBERTa sentiment model (https://huggingface.co/cardiffnlp/twitter-roberta-base-sentiment-latest)^51^. The model was trained on ~124 million tweets and fine-tuned using the TweetEval benchmark^52^. Our full analytical pipeline, from data collection to clustering and interpretation, is depicted in Fig. 1.

### Ethical considerations

We collected publicly accessible Reddit posts and comments between 2022 and 2024 using PRAW^42^ while adhering to ethical and legal frameworks, including international and national laws, Reddit’s Privacy Policy, and Subreddit Guidelines.

Health-related subreddits can serve as spaces for vulnerable groups, requiring researchers’ heightened ethical sensitivity to prevent reinforcing stigmatizing narratives^53^. Although Reddit posts are publicly visible, users typically use system-generated or self-chosen usernames instead of their real names. While sharing personally identifiable information about others is not allowed, there is still a risk that someone could be re-identified^53,54^. Direct quotations or shared context may risk re-identification through reverse searches, especially when personal information is embedded in users’ account histories.

To avoid reinforcing stigma and to safeguard participants’ privacy, we implemented additional measures for users’ data protection during data collection and processing. We did not collect data from any subreddits where the moderators, rules, or FAQs prohibit the use of data for research purposes. To further minimize traceability risks, no other profile information or metadata, such as user IDs was collected.

We oriented our de-identification process toward a previously published method^41^. User privacy was further protected through a two-step automated and manual de-identification pipeline. Personally identifiable information was removed using the GLiNER model^55^ by identifying and deleting named entities such as names, email addresses, and specific locations. User tags, physical addresses, telephone numbers, URLs, and other linked content (e.g., marked with “@”) were also removed. Direct quotations were paraphrased. In addition, any self-reported ages were categorized into broad categories (e.g., early twenties) rather than recorded as exact values. Moreover, the original usernames were replaced with anonymized identifiers. Posts and comments from users whose accounts had already been deleted before data collection were excluded from the analysis. A subsequent manual evaluation verified the efficacy of the anonymization procedures, and any remaining information we found that could facilitate identification was removed. Data were stored on secure, access-controlled internal servers with restricted project-team access.

## Results

### Classification and text passage extraction

After data collection and de-identification, posts and comments were aggregated into chunks at the user level. In total, 521,219 posts and comments were combined into 180,156 chunks. The chunks had a median word count of *Mdn* = 429 (*Q1* = 201; *Q3* = 917; *M* = 527.27; *SD* = 371.34). Of the 180,156 chunks, the LLM classified 5889 as related to psychotherapy dissatisfaction. This classification was validated by a human rater on a stratified random sample of 1000 chunks. Of the 500 chunks rated as “No” by the LLM, 469 (93.8%) were also rated as “No” by the human rater. Of the 500 chunks classified as “Yes”, 451 (90.2%) were confirmed as “Yes” by the human rater. This resulted in a Cohen’s Kappa of *κ* = 0.84, indicating high agreement between the LLM and the human rater.

The chunks classified as “Yes” by the LLM were further processed. Within these chunks, the LLM extracted 28,079 text passages related to psychotherapy dissatisfaction. A stratified random sample of 300 chunks was selected, in which a human rater, blind to the LLM-extracted passages, independently identified relevant text passages. We computed ROUGE scores to assess the agreement between the model and human-extracted text passages. The scores of ROUGE-1 = 0.68, ROUGE-2 = 0.58, and ROUGE-L = 0.68 indicate high content-level agreement between the LLM and the human annotator, suggesting that the automated extraction procedure performed well in capturing relevant text passages. The extracted text passages had a median word count of *Mdn* = 23 (*Q1* = 15; *Q3* = 33; *M* = 26,66; *SD* = 17,43) and were used for further analyses in this paper.

### Clustering and topic modeling

Using UMAP and HDBSCAN, the text passages were grouped into 55 clusters, with one cluster representing outliers. Internal metrics indicated good cluster quality (mean Silhouette = 0.41; Calinski-Harabasz = 15,391), and manual inspection confirmed semantic coherence. This configuration achieved the strongest validation scores and the most interpretable cluster structure among all parameter settings tested. For illustration, two de-identified examples of outlier text passages are “…my therapist didn’t lift a finger to help” and “After switching therapists, I began to feel that I was the problem and that therapy would never help”.

The cluster sizes varied significantly, ranging from 5657 (20.1%) to 25 (0.1%) associated text passages. Supplementary Table 2 presents an overview of the cluster names, assigned topics, and the number of associated text passages. The largest clusters, each covering more than 2% of text passages, were (1) Dissatisfaction with therapist behavior (20.1%), (2) Persistent therapy ineffectiveness (7.3%), (3) Difficulties in therapeutic alliance with therapist (6.8%), (4) Difficulties with past therapist fit and finding a suitable therapist (4.7%), (5) Perceived lack of progress (3.2%), (6) Negative feelings in therapy sessions (2.9%), and (7) Barriers to accessing mental health care (2.1%). Together, these clusters capture 47.1% of all dissatisfaction passages.

### Main themes

The identified clusters were mapped onto the qualitative framework developed in a recent meta-analysis^20^. In sum, 23.7% of text passages fell into the pre-determined cluster Therapists’ misbehavior, 16.9% into Negative impacts of treatment, 12.5% into Hindering aspects of the relationship, and 12.2% into Poor treatment fit. Additionally, we identified a new meta-category, Challenges in diagnostics and treatment of specific disorders/symptoms, which accounted for 6.9% of text passages and 12 clusters. This meta-category was assigned to the pre-determined cluster Poor treatment fit. We established a new cluster, Dissatisfaction with the health care system and costs, which comprises the two meta-categories Dissatisfaction with the health care system and Dissatisfaction with therapy costs, accounting for six clusters. This overarching cluster includes 3.9% of the text passages. Finally, 30.7% of text passages did not align with the pre-determined clusters.

The dissatisfaction captured in the Therapists’ misbehavior cluster primarily relates to inappropriate therapist behavior but also includes concerns about the therapist as a person, as well as accusations of invalidation, insensitivity, and incompetence. The most frequently mentioned issues in the Hindering aspects of the relationship cluster related to difficulties in the therapeutic relationship, past therapists who were not a good fit, and the challenge of finding a suitable therapist. Additionally, problems with open communication, avoidance of certain topics, and trust in the therapist played a significant role. The Poor treatment fit cluster captures frustrations with diagnostics and treatment for various mental health conditions (e.g., ADHD, borderline personality disorder), including modalities (CBT, EMDR, online therapy, inpatient treatment) and organizational failures such as abrupt therapist disengagement. Negative impacts of treatment reflect reports of long-term perceived lack of effectiveness in psychotherapy and harm, most often referring to psychotherapy in general, but in some cases also to specific therapeutic approaches. Additionally, many patients reported unpleasant emotions, while some mentioned experiencing negative thoughts and reluctance to re-engage in psychotherapy. Dissatisfaction with the health care system and costs highlights difficulties in accessing the mental health care system, problems finding suitable help, and generally negative experiences with the system. Additionally, high costs were described as a barrier to access, with some patients stating that the expenses were not justified by the perceived benefits of therapy. For detailed frequencies by cluster and meta-category, see Table 2.

**Table 2:** Pre-determined clusters

Cluster	Meta-category	Cluster label	Cluster number	*N t*ext passages
Therapists’ misbehavior (*n* = 6668, 23.7%)	Therapist not understanding (*n* = 375, 1.3%)	Not feeling heard and understood	12	375
	Therapist perceived as incompetent (*n* = 412, 1.5%)	Blaming the therapist	17	186
		Dissatisfaction with the psychologist	22	150
		Inadequate behavior of the therapist in anxiety treatment	44	38
		Perceived incompetence of professionals	45	38
	Therapist devaluing the client (*n* = 150, 0.5%)	Experiences of invalidation by a therapist in eating disorder treatment	21	150
	Other inappropriate verbal reactions (*n* = 5731, 20.4%)	Perceived insensitivity of therapists toward patients’ appearance concerns	51	32
		Dissatisfaction with therapist behavior	0	5657
		Perceived inadequate treatment of clients	43	42
Hindering aspects of the relationship (*n* = 3509; 12.5%)	Experiencing insecurity or distrust (*n* = 261, 0.9%)	Difficulties with open communication in therapy	20	167
		Trust issues regarding the therapist	40	51
		Avoidance of addressing difficult topics in therapy	42	43
	Experiencing poor interpersonal match with therapist (*n* = 1326, 4.7%)	Difficulties with past therapist fit and finding a suitable therapist	3	1326
	Experiencing distance and/or lack of empathy (*n* = 1922, 6.8%)	Difficulties in therapeutic alliance with the therapist	2	1922
Poor treatment fit (*n* = 3431, 12.2%)	Negative evaluation of practical aspects of therapy (*n* = 578, 1.2%)	Frustrations with scheduling, cancellations, and therapist accessibility	16	214
		Negative experiences with inpatient treatment	26	121
	Lack of fit with the intervention (*n* = 1127, 4.0%)	Dissatisfaction with CBT therapy	8	500
		Challenges in EMDR therapy	9	497
		Frustrations with IFS therapy	31	67
		Negative experiences in group therapy	52	28
		Dissatisfaction with online therapy	48	35
	Dissatisfaction with therapy ending (*n* = 34, 0.1%)	Experiences of being ghosted by therapists	49	34
	Challenges in diagnostics and treatment of specific disorder/symptoms (*n* = 1935; 6.9%)	Challenges in trauma therapy	10	471
		Challenges in eating disorder treatment	11	456
		Frustrations with the diagnostic process and treatment of autism	14	244
		Frustrations with the diagnostic process and treatment of ADHD	19	172
		Frustrations with the diagnostic process and treatment of borderline personality disorder	24	145
		Frustrations with the diagnostic process and treatment of OCD and OCPD	25	130
		Ineffective treatment of anxiety and panic attacks	29	78
		Struggles with treating dissociation	33	61
		Frustrations with the diagnostic process and treatment of CPTSD	34	59
		Ineffective treatment of sleep problems	41	49
		Frustrations with the diagnostic process and treatment of narcissistic personality disorder	47	36
		Frustrations with the diagnostic process and treatment of AVPD	50	34
Negative impacts of treatment (*n* = 4740, 16.9%)	No change or insufficient change (*n* = 3433, 12.2%)	Persistent therapy ineffectiveness	1	2039
		Perceived lack of progress	4	903
		Ineffectiveness and side effects of medication	13	284
		Ineffectiveness of talk therapy	28	94
		Ineffectiveness of mindfulness practices	35	59
		Struggles with the healing process	38	54
	Loss of motivation or hope; Resignation (*n* = 78, 0.3%)	Reluctance to resume therapy	30	78
	Unpleasant feelings during therapy (*n* = 995, 3.5%)	Negative feelings in therapy sessions	5	819
		Emotional responses to therapy	32	66
		Negative feelings during and after therapy sessions	37	59
		Overwhelming negative emotions in therapy sessions	39	51
	Negative cognitions arose in therapy (*n* = 234, 0.8%)	Negative thought patterns	15	234
Dissatisfaction with the health care system and costs (*n* = 1097; 3.9%)	Dissatisfaction with the health care system (*n* = 854; 3.0%)	Barriers to accessing mental health care	6	578
		Negative experiences in the mental healthcare system	27	98
		Not obtaining suitable help in the healthcare system	18	178
	Dissatisfaction with therapy costs (*n* = 243; 0.9%)	Financial barriers to therapy	23	147
		Frustrations with costs and the value of therapy	36	59
		Therapy costs are not worth it	46	37
No fit (*n* = 8634; 30.7%)		Dissatisfaction with the diagnostic process and psychiatrists	7	510
		Negative experiences with medical providers	53	25
		Outliers	−1	8099

The numbers in parentheses in the “Cluster” column represent the number of text passages associated with each reflective pre-determined cluster.

### User-level analysis

On average, each user contributed *M* = 5.24 (*SD* = 5.87) text passages related to psychotherapy dissatisfaction. These passages were distributed across *M* = 2.42 (*SD* = 1.63) of the newly generated clusters and *M* = 1.90 (*SD* = 0.95) of the pre-determined clusters. For a detailed distribution of the pre-determined clusters at the user level, see Table 3.

**Table 3:** Distribution of user text passages across pre-determined clusters

Cluster label	*N* text passages	Total users	Only this cluster (users)	Avg. co-occurring clusters (user)	Co-occurrences with other clusters (user)
Therapists’ misbehavior	6668	2219	514	1.33	Negative impacts of treatment (1057) Poor treatment fit (828) Hindering aspects of the relationship (704) Dissatisfaction with the healthcare system and costs (371)
Hindering aspects of the relationship	3509	1666	312	1.47	Negative impacts of treatment (814) Therapists’ misbehavior (704) Poor treatment fit (618) Dissatisfaction with the healthcare system and costs (276)
Poor treatment fit	3431	1962	377	1.42	Negative impacts of treatment (998) Therapists’ misbehavior (828) Hindering aspects of the relationship (618) Dissatisfaction with the healthcare system and costs (338)
Negative impacts of treatment	4740	2720	747	1.22	Therapists’ misbehavior (1,057) Poor treatment fit (998) Hindering aspects of the relationship (814) Dissatisfaction with the healthcare system and costs (403)
Dissatisfaction with the healthcare system and costs	1097	776	95	1.79	Negative impacts of treatment (403) Therapists’ misbehavior (371) Poor treatment fit (338) Hindering aspects of the relationship (276)

Negative impacts of treatment were the most common pre-determined cluster, appearing in 2720 users. It was also the most frequently occurring cluster without co-occurrence, appearing alone in 747 users. Furthermore, Negative impacts of treatment were the most frequently co-occurring cluster across all five other clusters, followed by Therapists’ misbehavior as the second most common co-occurrence. Thus, these clusters were the most frequently co-occurring pair, found together in 1057 users. Conversely, Dissatisfaction with the health care system and costs appeared in the fewest users (95) and was also the least frequently occurring in isolation. Additionally, it was the least frequently co-occurring cluster overall.

### Sentiment analysis

The mean negative sentiment across the newly generated clusters was *M* = 0.70 (*SD* = 0.06), while the mean neutral sentiment was *M* = 0.25 (*SD* = 0.05) and the mean positive sentiment was *M* = 0.05 (*SD* = 0.02). The five clusters most strongly associated with negative sentiment were: (1) Negative experiences with medical providers (*M* = 0.85); (2) Negative experiences in the mental healthcare system (*M* = 0.83); (3) Frustrations with costs and the perceived value of therapy (*M* = 0.79); (4) Frustrations with the diagnostic process and treatment of narcissistic personality disorder (*M* = 0.79); and (5) Overwhelming negative emotions in therapy sessions (*M* = 0.78).

Among the pre-determined clusters, Negative impacts of treatment exhibited the highest average negative sentiment (*M* = 0.73), followed by Dissatisfaction with the health care system and costs (*M* = 0.72) and Poor treatment fit (*M* = 0.69). Therapists’ misbehavior had the second-lowest negative sentiment, while Hindering aspects of the relationship had the lowest.

## Discussion

Negative experiences in psychotherapy are an underreported phenomenon, which makes it difficult to research with traditional approaches. To explore these experiences, we applied an innovative research approach by combining multiple advanced quantitative and qualitative methods to analyze large amounts of online forum posts related to psychotherapy dissatisfaction. We identified over 28,000 text passages that explicitly address dissatisfaction with psychotherapy, ~30 times more than the largest published study on this topic^20^. These covered a wide range of dissatisfaction themes, grouped into four overarching categories: Therapists’ misbehavior, Hindering aspects of the relationship, Poor treatment fit, and Negative impacts of treatment, as well as two additional themes not present in prior frameworks: Dissatisfaction with the health care system and costs, and Challenges in diagnostics and treatment.

Our study shows that dissatisfaction with psychotherapy is not rare or trivial but complex, emotionally charged, and often driven by unmet needs, poor therapeutic relationships, and systemic failure. We combined unsupervised NLP techniques with qualitative validation to explore this phenomenon, allowing for both scalable detection and interpretative depth. While unsupervised algorithms enabled efficient pattern recognition, reading representative text passages proved crucial for understanding nuanced dissatisfaction themes that topic modeling alone could not capture. Human interpretation remained essential for semantic refinement and accurate cluster labeling. This mixed-methods approach allowed us to combine the strengths of large-scale analysis with in-depth understanding.

Our results are consistent with prior findings identifying harmful therapist behavior and ruptured alliances as key drivers of dissatisfaction^26,27,29^. Patients reported feeling pressured, misunderstood, or invalidated. We also frequently encountered dissatisfaction, or even frustration, with the diagnosis and therapy of specific disorders (e.g., autism, eating disorders), and dissatisfaction with therapy methods. This included perceived misdiagnoses, unclear diagnostic communication, and frustration with therapy approaches that felt confusing or invalidating to patients. These findings underscore the importance of personalized approaches, including shared decision making, to enhance the fit and acceptance of treatment^56^. Most users expressed dissatisfaction across multiple domains, with frequent co-occurrence of clusters such as Negative impacts of treatment and Therapists’ misbehavior. This highlights that dissatisfaction is often multifaceted rather than limited to a single aspect of the therapeutic experience. The cluster Negative impacts of treatment is similar to previously described domains of adverse effects, particularly those involving intense negative emotions^26^. In the present analysis, this cluster exhibited the highest levels of negative sentiment. Notably, it also captured long-term dissatisfaction with therapy, sometimes reported years after treatment had concluded. This has not been documented in prior work^27^. In addition to interpersonal and process-related dissatisfaction, our analysis revealed broader structural concerns. We identified systemic barriers to accessing therapy, dissatisfaction with healthcare structures, and frustration about high treatment costs. These themes were associated with particularly strong negative sentiment, especially regarding medical providers and institutional failures. This suggests that dissatisfaction extends beyond the therapy room and reflects deeper systemic problems. By combining unsupervised NLP clustering with manual validation, our study fills important gaps in understanding the full scope of psychotherapy dissatisfaction from the patient’s perspective.

Our linguistic analyses revealed that many users frequently expressed negative affect, which was reflected in clusters such as “Frustrations with scheduling, cancellations, and therapist accessibility”. Sentiment analysis confirmed a strong expression of negative emotions, particularly in relation to perceived treatment failures, which aligns with prior findings in which emotional themes emerged as central components of client-identified hindering experiences in psychotherapy^27^. The anonymity of online forums likely encouraged more open self-disclosure than traditional research settings, enabling users to express dissatisfaction without fear of judgment or negative interpersonal consequences^31,32^. These results underscore the value of online forum data for capturing authentic patient perspectives in psychotherapy research.

In sum, our findings make visible a diverse and often overlooked spectrum of dissatisfaction that arises both from individual therapeutic interactions and broader systemic barriers. By listening to patients’ voices in online spaces, we uncover dimensions of dissatisfaction that may remain hidden in clinical settings and begin to close a major gap in psychotherapy research.

This study has several limitations that affect most work relying on self-reported experiences in online contexts^41^. While demographic information was limited to self-reports, we were still able to collect substantial sample data, ensuring strict data protection and user anonymity. The de-identification process, while essential for privacy policy, harmed data quality by modifying or removing certain information. However, a key priority in analyzing online forum posts is preserving user privacy through strict de-identification processes, as we did. This affected both the quality of sentence embeddings and the availability of sample-related data. The generalizability of our findings is limited due to the platform-specific sample. As our data are drawn from online forums, the findings reflect the perspectives of individuals who chose to share their experiences in these spaces. While this provides valuable access to otherwise underreported phenomena, it does not allow for conclusions about the absolute prevalence of such experiences in the general population. Although our sample is skewed toward younger, Western users, it included a substantial proportion of non-binary and neurodiverse individuals, offering valuable access to voices that remain underrepresented in traditional clinical research. Despite its platform-specific nature, the dataset spans a wide demographic spectrum, including users of diverse ages, national backgrounds, and diagnostic categories, thus offering a broader perspective than many conventional clinical samples. To maintain high cluster quality, a significant number of outlier text passages (8099) were excluded. These outliers were identified based on their statistical deviation from other data points. Since all text passages had previously been extracted by the LLM as relevant to psychotherapy dissatisfaction and verified through manual checks (with overlap calculated using ROUGE-scores), the majority of outliers were still related to the main theme of dissatisfaction. While this exclusion helped ensure the purity and coherence of the clusters, it also meant that some data were not included in the final analysis. Although these passages were statistically classified as outliers by HDBSCAN, a qualitative review suggested that some of them may be categorized thematically in one or more of the identified clusters, or could potentially form additional, smaller clusters if further subdivided.

Of the 55 newly generated clusters, the seven largest captured nearly half of all dissatisfaction passages. We mapped 52 of these clusters onto the pre-determined clusters and added one newly created cluster not represented in the meta-analysis, together covering more than two-thirds of all text passages. However, one third of the pre-determined meta-categories were not represented in the newly generated clusters (e.g., Therapist using client for own benefit), and some text passages addressed multiple themes. In such cases, passages were assigned to the most fitting meta-category. Although the quality of the clusters was maintained, further validation of the clustering process could have been conducted to enhance the robustness of the results. As our method is exploratory and data-driven, the number and granularity of clusters depend on choices of embeddings, dimensionality reduction techniques, and clustering parameters. We selected the solution that optimized interpretability and validation metrics, but using alternative algorithms and parameters remains possible. Due to the scope of this study, we were unable to include a comprehensive validation process within the current paper. Such additional validation could provide deeper insights into the reliability and stability of the identified clusters and would be a valuable next step in future research.

While exploratory clustering was appropriate for our study, as the aim was to discover new patterns in negative psychotherapy experiences, a hypothesis-driven classification approach may be more suitable for future research when predefined categories or theoretical frameworks exist. In such cases, the focus would shift towards testing existing theories or validating specific constructs rather than uncovering novel insights. Based on these results, a taxonomic model of patients’ negative experiences in psychotherapy should be developed and empirically tested. Specifically, the distinctiveness of the identified clusters or categories needs to be examined to determine whether patients’ statements can be reliably classified. Additionally, quantitative research is warranted to assess the frequency with which these dimensions appear across different samples.

Future studies should not only address the theme of patients’ dissatisfaction but also consider its duration and intensity. It remains unclear whether observable indicators of the clusters of negative psychotherapy experiences identified in our study emerge during therapy, and if so, at which stage of the therapeutic process these signs become apparent. Also, it is essential to investigate how the identified clusters relate to poorer treatment outcomes, particularly in connection with the therapeutic relationship and therapy dropout. Personalized approaches that address the unique needs of patients are essential for effective clinical practice. Therapists should be aware that certain individual behavior patterns can evoke significant negative reactions in patients. Moreover, establishing a profound therapeutic alliance is crucial to fostering trust and open communication. Additionally, feedback systems can support therapists in identifying and addressing patients’ negative experiences. Given the considerable dissatisfaction with the healthcare system and high treatment costs, technological advancements such as LLM-based therapy may offer a future option, though their impact on patient satisfaction remains uncertain.

Our study presented an innovative approach to analyzing huge amounts of mental health text data, focusing on negative experiences. By translating these insights into practical tools and theoretical frameworks, we can advance more responsive, patient-centered psychotherapy. We hope our findings contribute to a better understanding of these experiences and encourage future research to apply similar methods to improve patient outcomes.

## Supplementary information

Supplementary Information

## Data Availability

In the interest of full transparency and reproducibility, we have made our full analysis pipeline publicly accessible at OSF (https://osf.io/3uw8r/?view_only=8cf0708f2eaa44478a1eca3f22734c82), including all preprocessing, de-identification, and further analysis scripts. We also supply a complete list of Reddit posts and comments IDs, which allows qualified researchers to re-download the original data via the PRAW API under Reddit’s standard terms. In addition, we share the aggregated, fully anonymized outputs (for example, cluster assignments) so that every step of our workflow can be inspected and replicated. Although verbatim user posts cannot be redistributed due to our ethical approvals, GDPR compliance, and Reddit’s Terms of Service, these combined resources provide everything needed to reproduce and extend our findings.
