# Supplementary material for: Exploring negative experiences in psychotherapy using an NLP approach on online forum data
Source: Npj Ment Health Res. 2025 Nov 7;4:59. doi: 10.1038/s44184-025-00172-4 (PMC12592706; doi:10.1038/s44184-025-00172-4)
Supplement: Supplementary file 1 — Supplementary Information [file 44184_2025_172_MOESM1_ESM.docx]

**Supplementary Table 1|Overview of clustering procedure and hyperparameters**

| Step | Method | Key Parameters | Purpose |
| --- | --- | --- | --- |
| 1. Embedding generation | SentenceTransformer model all-mpnet-base-v2 | Default settings | Create contextual embeddings for each text passage |
| 2. Dimensionality reduction | UMAP | n_neighbors = 5; n_components = 5; min_dist = 0.02; metric = “cosine”; random_state = 42 | Improve cluster quality |
| 3. Clustering | HDBSCAN | min_cluster_size = 20, min_samples = 30, cluster_selection_epsilon = 0.28 | Identify dense clusters; allow flexible cluster size; Text passages not assigned to any cluster due to insufficient local density were considered outliers |
| 4. Cluster validation | Silhouette score  Calinski-Harabasz Index  Brief manual inspection (two raters) |  | Assessment of intra-cluster cohesion vs. inter-cluster separation  Comparison of between-cluster and within-cluster variance  Verification of semantic cluster coherence |
| 5. Cluster labeling | Manual Review (two raters) | Guided by principles of thematic analysis; supported by BERTopic; discussing and reaching consensus of final cluster names | Refine and label clusters |

The silhouette score compares the distance of a data point (in our case, the embedding of a text passage) to other points within the same cluster with its distance to points in the nearest neighboring cluster. A mean silhouette value of 0 indicates overlapping clusters, whereas a value of 1 signifies high intra-cluster similarity and clear separation from other clusters. The Calinski-Harabasz index compares the variance between clusters to the variance within clusters. A higher value indicates better cluster quality.

**Supplementary Table 2|Newly generated clusters and topics**

| Num-ber | Name | Topics | Text passages | |
| --- | --- | --- | --- | --- |
|  |  |  | Number | % |
| 0 | Dissatisfaction with therapist behavior | said, told, asked, session, like, felt, saying, think, way, feel | 5,657 | 20.1 |
| 1 | Persistent therapy ineffectiveness | therapy, therapy not, years, going therapy, treatment, not help, therapy not help, tried therapy, therapy years, help | 2,039 | 7.3 |
| 2 | Difficulties in therapeutic alliance with therapist | said, told, saying, asked, say, he would, guy, things, tell, session | 1,922 | 6.8 |
| 3 | Difficulties with past therapist fit and finding a suitable therapist | therapists, therapist, new therapist, new, therapist not, good, therapists not, fit, therapists just, good therapist | 1,326 | 4.7 |
| 4 | Perceived lack of progress | progress, feel, feel like, feels, really, feels like, felt, worse, like, helped | 903 | 3.2 |
| 5 | Negative feelings in therapy sessions | session, sessions, feeling, felt, left, session feeling, talk, left feeling, week, feel | 819 | 2.9 |
| 6 | Barriers to accessing mental health care | insurance, appointment, appointments, provider, staff, insurance not, afford, paying, providers, pay | 578 | 2.1 |
| 7 | Dissatisfaction with diagnostic process and psychiatrists | psychiatrist, diagnosis, bipolar, psychiatrists, psychiatrist not, psych, symptoms, diagnosed, new psychiatrist, diagnoses | 510 | 1.8 |
| 8 | Dissatisfaction with CBT therapy | cbt, dbt, cbt not, cbt therapist, like cbt, cbt dbt, dbt not, tried cbt, cbt therapy, doing cbt | 500 | 1.8 |
| 9 | Challenges in EMDR therapy | emdr, erp, emdr therapist, emdr therapy, doing emdr, emdr not, tried emdr, started emdr, emdr months, emdr session | 497 | 1.8 |
| 10 | Challenges in trauma therapy | trauma, ptsd, trauma not, trauma therapist, trauma therapy, traumatized, abuse, traumatic, trauma just, childhood trauma | 471 | 1.7 |
| 11 | Challenges in eating disorder treatment | eating, weight, ed, eating disorder, eat, food, anorexia, binge, disorder, meal | 456 | 1.6 |
| 12 | Not feeling heard and understood | christmas, say, tell, wedding, understand, heard, matter, christmas eve, eve, ask | 375 | 1.3 |
| 13 | Ineffectiveness and side effects of medication | meds, medication, dose, effects, taking, seroquel, antidepressants, zoloft, pills, prescribed | 284 | 1.0 |
| 14 | Frustrations with diagnostic process and treatment of autism | autism, autistic, autistic people, not autistic, autism not, aba, people, adults, adhd, autistic person | 244 | 0.9 |
| 15 | Negative thought patterns | thoughts, positive, negative, accept, change, self, feel feelings, things, guilty, parts | 234 | 0.8 |
| 16 | Frustrations with scheduling, cancellations, and therapist accessibility | terminated, fired, reschedule, appointment, cancel, schedule, appointments, late, cancellation, 72hour | 214 | 0.8 |
| 17 | Blaming the therapist | therapist, therapist not, therapist therapist, maybe therapist, maybe therapist not, misunderstanding, mistakes, experience therapist, reaction, strike | 186 | 0.7 |
| 18 | Not obtaining suitable help in healthcare system | support, help, help need, trying help, getting help, need, got support, need support, reaching help, getting | 178 | 0.6 |
| 19 | Frustrations with diagnostic process and treatment of ADHD | adhd, adhd diagnosis, not adhd, diagnosed adhd, believe adhd, adhd not, assessment, adhd medication, diagnosed, not believe adhd | 172 | 0.6 |
| 20 | Difficulties with open communication in therapy | open, opening, honest, honest therapist, talk, therapist asks, fully honest, hard open, conversation therapist not, therapist not talk | 167 | 0.6 |
| 21 | Experiences of invalidation by therapist in eating disorder treatment | eating, weight, food, binge, eat, eating disorder, binge eating, anorexic, underweight, lose weight | 150 | 0.5 |
| 22 | Dissatisfaction with psychologist | psychologist, psychologists, psychologist not, psychology, psych, seeing psychologist, psychologists feel, let talk current, ignored thatis definitely, going work leave | 150 | 0.5 |
| 23 | Financial barriers to therapy | insurance, afford, pay, pocket, cost, cover, takes insurance, pay pocket, insurance not, expensive | 147 | 0.5 |
| 24 | Frustrations with diagnostic process and treatment of borderline personality disorder | bpd, bpd diagnosis, told bpd, bpd not, diagnosis, mom bpd, personality, disorder, like bpd, bpd symptoms | 145 | 0.5 |
| 25 | Frustrations with diagnostic process and treatment of OCD and OCPD | ocd, ocd specialist, ocd not, therapy ocd, treating ocd, nocd, not ocd, specialist, ocd therapy, ocpd | 130 | 0.5 |
| 26 | Negative experiences with inpatient treatment | hospital, facility, send, forced, send away, mental hospital, discharge, harming, rights, staff | 121 | 0.4 |
| 27 | Negative experiences in mental healthcare system | mental health, health, mental, nhs, health professionals, mental health professionals, mental health services, health services, professionals, mental healthcare | 98 | 0.3 |
| 28 | Ineffectiveness of talk therapy | talk therapy, talk, talk therapy not, talk therapy years, talk therapy just, tried talk therapy, feel typical talk, feel typical, typical talk, typical talk therapy | 94 | 0.3 |
| 29 | Ineffective treatment of anxiety and panic attacks | anxiety, panic, attacks, panic attacks, anxiety just, not reducing, having panic attacks, reducing, having panic, therapist intense fear | 78 | 0.3 |
| 30 | Reluctance to resume therapy | want not think, needless say, needless, not look forward, going, look forward, not want not, horrible experience, money attend, not point bothered | 78 | 0.3 |
| 31 | Frustrations with IFS therapy | ifs, ifs therapist, ifs therapy, started ifs, ifs not, parts, doing ifs, ifs institute, not ifs, institute | 67 | 0.2 |
| 32 | Emotional responses to therapy | edge, invalidation, studies, distress, worsened, leaning positive psychological, no thoughts dissociated, no longer able, news possibly totally, news possibly | 66 | 0.2 |
| 33 | Struggles with treating dissociation | dissociation, dissociate, dissociated, dissociation just, dissociating, breathing mindfulness, talk dissociation, poem, knowledge dissociation, experience knowledge | 61 | 0.2 |
| 34 | Frustrations with diagnostic process and treatment of CPTSD | cptsd, cptsd not, cptsd years, citpd, tell cptsd, having cptsd, distressing times, compassion therapists, threat life, compassion therapists work | 59 | 0.2 |
| 35 | Ineffectiveness of mindfulness practices | meditation, grounding, mindfulness, exercises, techniques, grounding exercises, breathing, grounding techniques, exercises not, breathing exercises | 59 | 0.2 |
| 36 | Frustrations with costs and value of therapy | waste, money, time money, waste time money, waste money, waste time, hated, yes waste, money yes, money energy | 59 | 0.2 |
| 37 | Negative feelings during and after therapy sessions | therapy session, therapy session left, session cause distress, session cause, therapy session cause, session left broken, left broken feels, know therapy session, broken feels, feels like ptsd | 59 | 0.2 |
| 38 | Struggles with the healing process | healing, heal, healed, exiles, missing recovery, reason scared, protectors, therapist key, repairing, bond | 54 | 0.2 |
| 39 | Overwhelming negative emotions in therapy sessions | crying, really really really, really really, yelled, cried, really felt used, felt used, days just bed, know express really, just bed not | 51 | 0.2 |
| 40 | Trust issues regarding the therapist | trust, trust therapists, trust therapist, trust therapists anymore, therapists anymore, not trust therapist, not trust, trust gone, trust therapist anymore, longer feel trust | 51 | 0.2 |
| 41 | Ineffective treatment of sleep problems | sleep, bed, sleeping, sleep hygiene, sleep not, hygiene, melatonin, sleep bed, sleep studies, psychiatry repeatedly fails | 49 | 0.2 |
| 42 | Avoidance of addressing difficult topics in therapy | bring, past think, well meaning, bring therapist, makes uncomfortable, ask therapist, not told psychologist, open touch, occur not realize, open therapist not | 43 | 0.2 |
| 43 | Perceived inadequate treatment of clients | client, clients, they would leaving, client service, client not, service, just client, minimizing, company, client abandonment | 42 | 0.1 |
| 44 | Inadequate behavior of therapist in anxiety treatment | anxiety, talking anxiety, stop talking anxiety, anxiety focusing, refused really, refused really address, therapist refused really, address issue told, really address issue, progress anxiety | 38 | 0.1 |
| 45 | Perceived incompetence of professionals | professionals, expert, supposed expert, professional, experts, unlikely, entitled, field, point entire, people man discouraging | 38 | 0.1 |
| 46 | Therapy costs not worth it | money, loop worries anxiety, expensive unemployed cost, wasting money run, worries anxiety, worries anxiety not, cost therapy generated, work wasting money, generated, plus expensive unemployed | 37 | 0.1 |
| 47 | Frustrations with diagnostic process and treatment of narcissistic personality disorder | narcissistic, narc, narcissism, traits, narcissistic traits, narcs, covert, narcissist, npd, narc abuse | 36 | 0.1 |
| 48 | Dissatisfaction with online therapy | online, telehealth, online therapy, online therapists, online therapies, fan felt distant, highly preferred, fan felt, therapies not massive, preferred face face | 35 | 0.1 |
| 49 | Experiences of being ghosted by therapist | ghosted, therapist ghosted, ghosted therapist, ghost, ghosted therapist ghosted, ghosting, months ago felt, years therapeutic, multiple times fucking, middle virtual therapy | 34 | 0.1 |
| 50 | Frustrations with diagnostic process and treatment of AVPD | avpd, avpd not, not avpd, diagnosed avpd, avpd makes, diagnosis avpd, avpd diagnosis, bpd avpd, not avoid, constantly trying | 34 | 0.1 |
| 51 | Perceived insensitivity of therapists toward patients’ appearance concerns | appearance, dysmorphia, body, body dysmorphia, body image, image, photo, ugly, photos, height | 32 | 0.1 |
| 52 | Negative experiences in group therapy | group, group therapy, going group, going group therapy, group feel like, not group, group not, group sessions, group feel, story not | 28 | 0.1 |
| 53 | Negative experiences with medical providers | disease, feel better disease, better disease, doctors, hates, taken help treatment, knows js, knows js makes, talk quarter, yes went years | 25 | 0.1 |
| -1 | Outliers | therapist, therapists, feel, help, just, therapy, no, really, know, therapist not | 8,099 | 28.8 |
| Total | - | - | 28,079 | 100.0 |

The numbers represent the original cluster numbers generated in Python. For each topic, the ten most frequent n-grams are listed.

**Supplementary Note 1|Instruction prompt for classification of personal psychotherapy dissatisfaction**

prompt_template = """
 ### INSTRUCTION:
Your task is to classify online forum posts to determine if they relate to "Personal Psychotherapy Dissatisfaction."
Provide the response for each post as either "Yes" or "No" only. Please adhere to the provided definition, examples, and criteria when making your classification. Additionally, provide a brief explanation for your decision, summarizing the key factors or phrases in the post that led to your conclusion with a maximum of 20 words.

### DEFINITION:
"Personal Psychotherapy Dissatisfaction" is defined as a negative experience or a feeling of being dissatisfied with one's own personal psychotherapy experience.
This negative experience may relate to the therapy setting, the therapist (e.g., characteristics or behaviors of the therapist), the therapeutic process, the therapeutic approach, the patient’s behavior in therapy, patient stagnation or deterioration, costs, or access to personal psychotherapy. Posts must clearly indicate dissatisfaction with **one's own personal experience in therapy** to be classified as "Yes."

### IMPORTANT NOTE:
Posts discussing dissatisfaction in general, therapy advice for others, or unrelated topics (e.g., childhood trauma, general life difficulties, relationship issues, abuse) **must** be classified as "No" unless they explicitly mention dissatisfaction with the user's **own psychotherapy** experience.
A connection to the user's own therapy must be **explicitly mentioned or at least clearly implied** in the post. If this connection is not evident, classify the post as "No."
Posts that express **general frustration or dissatisfaction, relationship difficulties, or emotional pain** without explicitly or clearly implying a connection to the user's own psychotherapy **must** be classified as "No."
Negative language or strong emotional expressions alone are not enough to classify a post as "Yes."

 ### CRITERIA:
**First person pronouns** (e.g., I, me, my) referring to the person's own therapy.
**Dissatisfaction** with the user’s own psychotherapy experience.

 ### EXAMPLES OF PERSONAL PSYCHOTHERAPY DISSATISFACTION ("Yes"):
- I am dissatisfied with the progress I made so far in my therapy.
- My therapist cannot understand me during my therapy sessions.
- I am so frustrated with my therapist; he never listens to what I say.
- My therapy sessions make me feel worse instead of better.
- The cost of my therapy is too high, and I don’t feel it's worth it.

 ### EXAMPLES OF NOT RELATED TO PERSONAL PSYCHOTHERAPY DISSATISFACTION ("No"):
- If I were you, I would look for a new therapist. (General advice, not personal dissatisfaction)
- I had a difficult childhood and don’t trust people easily. (Childhood issues but no mention of personal therapy dissatisfaction)
- My therapy sessions are going well, and I am making steady progress. (Positive experience)
- Therapy in general is too expensive for most people. (General statement, no personal dissatisfaction)
- My partner treats me horribly, and I don't know what to do. (Relationship issues, not therapy dissatisfaction)
"""

**Supplementary Note 2|LLM extraction prompt for personal psychotherapy dissatisfaction passages**

prompt_template = """
### INSTRUCTION:
The following chunk has been classified as "Yes" for Personal Psychotherapy Dissatisfaction: "{chunk}".
Your task is to extract **only the specific text passages** that directly indicate dissatisfaction with the user's own
psychotherapy experience.

### DEFINITION:
"Personal Psychotherapy Dissatisfaction" is defined as a negative experience or a feeling of being dissatisfied with
one's own personal psychotherapy experience. This negative experience may relate to the therapy setting, the therapist
(e.g., characteristics or behaviors), the therapeutic process, the therapeutic approach, the patient’s behavior in
therapy, patient stagnation or deterioration, costs, or access to personal psychotherapy. The extracted text must
clearly indicate dissatisfaction with **the user's own personal therapy experience**.

### IMPORTANT NOTE:
- Only include text passages that explicitly mention dissatisfaction with the user's **own psychotherapy** experience.
- Exclude statements that focus on external factors (e.g., family, friends, general life issues) unless they are directly tied to dissatisfaction with therapy.
- General emotional expressions (e.g., frustration, sadness) or unrelated complaints (e.g., relationship issues, childhood trauma) **must not** be included.
- If a text passage contains both relevant and irrelevant statements, include **only the relevant parts**.

### CRITERIA:
- Look for **first-person pronouns** (e.g., I, me, my) indicating the user's own therapy.
- Extract **only phrases or sentences** that specifically mention dissatisfaction with the user's personal psychotherapy experience.

### EXAMPLES OF EXTRACTED TEXT PASSAGES:
**Yes (relevant passages):**
1. "I am dissatisfied with the progress I made so far in my therapy."
2. "My therapist cannot understand me during my therapy sessions."
3. "I am so frustrated with my therapist; he never listens to what I say."
4. "My therapy sessions make me feel worse instead of better."
5. "The cost of my therapy is too high, and I don’t feel it's worth it."

**No (irrelevant passages):**
1. "I had a difficult childhood and don’t trust people easily." (Childhood issues but no mention of personal therapy dissatisfaction)
2. "My partner treats me horribly, and I don't know what to do." (Relationship issues, not therapy dissatisfaction)
3. "I’ve tried lots of things but I end up not having enough motivation to consistently do it or I just can't get out of bed." (General frustration, no direct therapy mention)
4. "I just want my old life back." (General emotional expression, no mention of therapy)
5. "Therapy in general is too expensive for most people." (General statement, no personal dissatisfaction)

### FORMAT:
**Extracted text passages:**
1. <First relevant text passage>
2. <Second relevant text passage>
...

**Number of extracted text passages:** <Number of passages>
"""
